# Supplementary material for: Germline CSF3R Variant in Chronic Myelomonocytic Leukemia: Linking Genetic Predisposition to Uncommon Hemorrhagic Symptoms
Source: Int J Mol Sci. 2023 Nov 7;24(22):16021. doi: 10.3390/ijms242216021 (PMC10671365; doi:10.3390/ijms242216021)
Supplement: Supplementary file 1 [file ijms-24-16021-s001.zip › ijms-2650014-supplementary.pdf]

Supplementary Materials File S1

Sample collection and isolation of mononuclear cells

Peripheral blood and bone marrow samples were collected in EDTA tubes for sequencing analysis, and SODIUM CITRATE tubes for cytofluorimetric and coagulation analysis. A HEPARIN tube was also collected as positive control of platelet activation. Bone marrow mononuclear cells (BMMCs) and peripheral blood mononuclear cells (PBMCs) were isolated from EDTA tubes by Ficoll density-gradient using Lymphosep (Biowest) and lysed in RLT buffer (Qiagen, Ltd) additioned of 1% ß-mercaptoethanol. A fraction of cells was cryopreserved.

Rotational thromboelastometry (ROTEM)

Haemostatic data were recorded and analyzed with a ROTEM delta thromboelastometry analyzer (Werfen). INTEM, EXTEM, FIBTEM and APTEM assays were performed and coagulation time (CT, min), Clotting Formation Time (CFT, min), A5, A10, A20 (mm), Maximum Clot Firmness (MCF, mm) and Maximum Lysis (ML, %) were collected.

T cell isolation

PBMCs and BMMCs were thawed and human T cells were isolated by depletion of $CD14^+$, $CD15^+$, $CD16^+$, $CD19^+$, $CD34^+$, $CD36^+$, $CD56^+$, $CD123^+$ and $CD235a^+$, using the pan T cell isolation kit (Miltenyi Biotec), according to the manufacturer's instructions. Positive and negative fractions were lysed in TRIzol™ reagent.

Genomic DNA isolation

DNA was extracted from RLT + 1% ß-mercaptoethanol lysates using the Maxwell® RSC Blood DNA Kit (Promega Corporation) according to the manufacturer's instructions. DNA was obtained from cells lysed in TRIzol™ reagent (Invitrogen) according to the manufacturer user guide.

Next Generation Sequencing and variant calling

The mutational profile was determined using SOPHiA Myeloid Solution™ (SOPHiA GENETICS) as previously described [1].

CSF3R exon 17 amplification and Sanger sequencing

CSF3R E808K (c.2422G>A) mutation was confirmed by specific sequencing reactions. A total amount of 50 ng of DNA was used to perform polymerase chain reaction (PCR) with the FastStart High Fidelity PCR System and a ready-to-use solution of PCR grade nucleotides (Roche) and the following primers: FW: 5'-CAGCCCCAAGTCCTATGAGA-3' and REV: 5'-CCTCTTCTCCAGCTAGCTCA-3'. The following PCR thermal profile was used: 5 minutes at 95°C; 35 cycles as follow: 30 seconds at 95°C, 30 seconds at 58°C and 30 seconds at 72°C; 7 minutes at 72°C and a hold at 4°C.

PCR amplicons were purified using QIAquick PCR Purification Kit (QIAGEN) according to manufacturer's instructions. Samples were sequenced according to dideoxy procedure BigDye® Terminator v3.1 Cycle Sequencing Kit (Thermo Fisher Scientific) on a 3130 Series Genetic Analyzer (Thermo Fisher Scientific). Sequences were analyzed using GeneMapper™ Software 5 (Thermo Fisher Scientific).

Reference

1. Napolitano, R.; De Matteis, S.; Carloni, S.; Bruno, S.; Abbati, G.; Capelli, L.; Ghetti, M.; Bochicchio, M.; Liverani, C.; Mercatali, L.; et al. Kevetrin induces apoptosis in TP53 wild-type and mutant acute myeloid leukemia cells. *Oncol. Rep.* **2020**, *44*, 1561–1573. https://doi.org/10.3892/or.2020.7730.
